# Evolution of CrC_x_ Ceramic Induced by Laser Direct Energy Deposition Multilayered Gradient Ni204-dr60 Coating

**DOI:** 10.3390/ma16216865

**Published:** 2023-10-26

**Authors:** Yu Zhao, Ruobing Wang, Jian Zhang, Muhammad Imran Farid, Wenzheng Wu, Tianbiao Yu

**Affiliations:** 1 School of Mechanical and Aerospace Engineering, Jilin University, Changchun 130025, China; yzhao0109@jlu.edu.cn (Y.Z.); wrb12182023@163.com (R.W.); 13065201199@163.com (J.Z.); farid9919@mails.jlu.edu.cn (M.I.F.); 2 Chongqing Research Institute, Jilin University, Chongqing 401133, China; 3 School of Mechanical Engineering and Automation, Northeastern University, Shenyang 110819, China; tianbiaoyudyx@gmail.com

**Keywords:** laser direct energy deposition, Ni204–dr60 multilayered gradient coating, microstructure evolution, microhardness

## Abstract

The manufacturing process for many large components of machines leads to a difference in their properties and performances based on changes in location. Functionally graded materials can meet these requirements and address the issue of generation and expansion of interface cracks. Ni204–dr60 gradient coatings were successfully fabricated using laser direct energy deposition (LDED). Microstructure mechanism evolution and microhardness of the gradient coating were comprehensively investigated. The change in the precipitated phase at the grain boundary and the intergranular zones resulted in a change in microstructural characteristics and also affected the microhardness distribution. The reinforced phase of the Ni204 → dr60 gradient zone from Ni204 to dr60 gradually precipitated and was rich in Mo and Nb phase, lath-shaped CrC_x_ phase, network-shaped CrC_x_ phase, block shape (Ni, Si) (C, B) phase, block CrC_x_ phase, and block Cr (B, C) phase. The gradient coating thus acts as a potential candidate to effectively solve the problem of crack generation at the interface of dr60 and the substrate.

## 1. Introduction

Widespread and progressive development of the remanufacturing process has become the most requisite part of the advancement. Laser direct energy deposition (LDED) technology offers some fascinating characteristics, such as die-less manufacturing, short production cycle, and excellent laser-cladded performance. Relying on the three-dimensional model of parts, the laser trajectory is controlled to manufacture parts, showing high flexibility and a tendency to remain unaffected by the complexity of the parts [1,2,3,4,5,6,7]. Therefore, LDED technology was found to be favorable for enhancing recycling benefits, resulting in waste parts by enabling component refurbishment, thus gradually increasing its share in the remanufacturing industry [8]. Yu et al. [9] used additive the metal-layer deposition technique and directly deposited two specific types of metal powders, AISI-P21 and AISI-H13, onto the FC300, respectively. Zhang et al. [10] investigated the effect of building strategies on remanufacturing cylindrical parts using laser metal deposition. Notably, the hardness and toughness of castings and forgings such as gears and spindles exhibited various distribution patterns from outside to inside. Thus, laser repair using a single material cannot meet the requirements. Moreover, the quality of the interface between the LDED zone and the pending-repair component affects the remanufacturing quality and service life. Therefore, improvements in metallurgical bonding strength in interface zones, gradient of microhardness distribution, and surface performance constitute the key elements of LDED application in the field of remanufacturing. To solve the issues, gradient coating, composite materials, and parameter optimization have frequently been taken into account. It is worth noting that waste components with different degrees of defects increase material and coating performance requirements. Nevertheless, physical differences between component property and LDED material, which meet the performance requirements, are responsible for laser repair quality. Therefore, the property gradient distribution of laser repair coatings has become a focus of research [11].

Li et al. [12] used a carbon dioxide (CO_2_) laser to weld the dissimilar metals Inconel 625 and SUS 304. Element segregation in the inter-dendritic region was found to be responsible for the reduced tensile strength and toughness. Lee et al. [13] obtained similar results in the course of laser welding of the dissimilar metal alloys 690 and SUS 304L. They pointed out that the distribution of elements in the fusion zone near SUS 304L was not homogeneous. Nb was primarily distributed in the inter-dendritic zone; however, its distribution throughout the fusion zone was not homogeneous. Although fusion welding can join similar and dissimilar metals, the different coefficients of thermal expansion, element segregation, cracks, pores, and generation of brittle intermetallic phases are still the main factors affecting laser welding quality [14]. Defects and irregular compositional distribution in the fusion zone are still encountered, leading to the failure of the welded component. The use of functional gradient materials has proven to be an effective way of improving metallurgical bonding quality and performance gradient distribution [15,16,17].

Shah et al. [18] produced SS316L and Inconel 718 functionally graded material using laser direct metal deposition. The distribution of hardness in the sample exhibited an approximate parabolic curve that was not evident. Carroll et al. [19] fabricated a specimen from 304L stainless steel incrementally graded to Inconel 625, which had gradients of hardness and density. Secondary phases primarily emerged in the gradient zone and were found to be responsible for the undesirable properties. Wang et al. [20] characterized the gradient microstructure fusion interface of dissimilar metals between high Cr steel and alloy 617 (Ni-based alloy). The results revealed that the formation of the gradient microstructure was determined by the angle between the tangents of the fusion line and the interface of adjacent layers. Savitha et al. [21] prepared discrete and compositional graded samples of SS316 and Inconel 625 alloys using laser-wise deposition and appraised the properties of the interface region. The microhardness of discrete composite coatings containing Inconel 625 alloys ranging from 25 to 75% exhibited small variations in their values.

According to previous studies, functional gradient materials show good performance, can improve the strength of the joint region, and effectively meet the various requirements of the component. The graded materials can reduce the probability of defects induced by different physical parameters of dissimilar materials. Compared to ultrasonic welding and friction stir welding, the widely graded structure can solve the issues of large thermal and mechanical residual stresses resulting from the connection of dissimilar materials [16,22]. Direct laser fabrication is an ideal technology for preparing functional gradient materials. However, cracks still emerge easily in the interface between different materials. The combination of the quality between dissimilar materials still meets the diversity requirements, albeit with difficulties.

Nickel (Ni)-based alloys are one of the most widely used metal alloys with good self-embellishment [23]. Although numerous studies are available on Ni-based alloy coatings, only a few studies on Ni–Ni gradient coatings have been reported to date. According to our previous study, the Ni204 alloy has good wettability with 45 steel, good toughness, and corrosion resistance, but low hardness [24]. Dr60 alloys (Nickel-based alloys) have high microhardness that can meet the requirements of machine components; nonetheless, the poor toughness and large difference in physical property parameters lead to bad bonding quality and a high probability of crack generation [25,26]. Therefore, corrosion-resistant Ni204 alloy and dr60 alloy were selected herein to study the effect of Ni204-based alloy as a transition material between different types of high-hardness powders (Ni-based) and substrates on coating properties. These two alloys primarily contain Fe, Ni, and Cr elements, which have good solubility. The fabrication of graded materials from Ni-based alloy to stainless steel has been previously studied [18,27,28]. These selected materials can effectively meet the criteria for wrought components of high microhardness and wear resistance, such as gear or spindle, although reduced toughness and defects, including cracks and uneven hardness gradient distribution, still exist. Therefore, functional gradient materials can be considered effective alternatives for laser repair of large, damaged parts. Functional gradient Ni204–dr60 materials were reported in this study for the first time, while previous studies were mainly focused on process optimization [29].

In this study, the discrete layer Ni204_X_dr60_Y_ (0 ≤ x ≤ 80%, 20% ≤ y ≤ 100%, and x + y = 1, x and y stand for weight percent) and Ni204–dr60 functional gradient materials were fabricated using LDED by varying the composition of the powder between layers to obtain a hardness gradient. The microstructure of CrC_x_ ceramic, the elemental composition, and the microhardness of discrete and compositional materials with graded Ni204–dr60 coatings were subjected to contrastive analyses. Additionally, the relationship between CrC_x_ ceramic content and the characteristic and hardness gradient distribution was systematically investigated.

## 2. Experimental Section

### 2.1. Materials and Equipment

The substrate material employed in this study is 45^#^ steel. Ni204 (Zhongke Yuchen (Beijing, China), spherical, 53 μm < particle size < 150 μm) and dr60 (Stellite, spherical, 53 μm < particle size < 150 μm) powders were used as initial materials for the fabrication of Ni204–dr60 gradient samples. Table 1 gives the chemical compositions of the powders and substrate used.

The open-loop control laser direct energy deposition (LDED) system was employed to fabricate the gradient materials. The IPG fiber (YLR-500W), with a wavelength of 1020 nm, was employed in the LDED experiments. Pure argon (99.99%) was used as the shielding gas and powder-sending gas. A laser power of 450 W with 1.1 mm diameter spots, a scan speed of 5.5 mm s^–1^, and a powder sending rate of 0.7 r min^–1^ were used as the main LDED parameters. In this study, discrete layers of Ni204_X_dr60_Y_ (20% ≤ Y ≤ 90%, X + Y = 1) and gradient samples consisting of 10 different layers (as presented in Table 2) with compositions ranging from pure Ni204 to pure dr60 were prepared. Before the experiment was conducted, the cladding powder was homogeneously mixed using Al_2_O_3_ ball milling at 30 rpm for 2 h to reduce segregation and then dried in a drying oven at 120 ℃ for 6 h to remove moisture.

**Figure 1:**
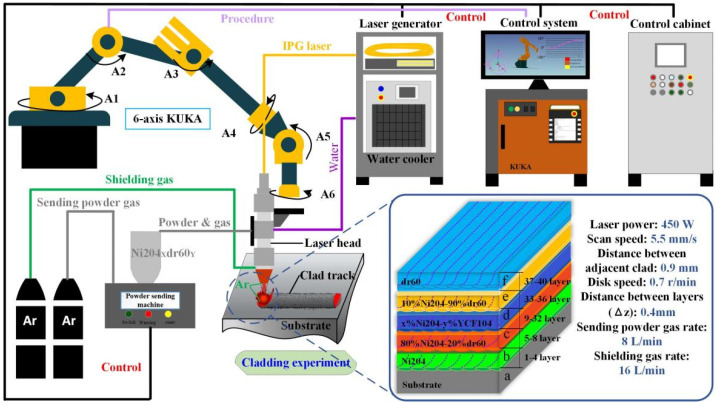
Schematic illustration of gradient fabrication.

Figure 1 shows a schematic illustration of the equipment, forming path, and forming mechanism. The program switch obtained using the off-line programming software-controlled KUKA robot drives the LDED head to move and realize the LDED part formation [30]. The distances between the two layers and the adjacent clad were 0.4 mm and 0.9 mm, respectively. To avoid oxidation and guarantee the bunching of the powder, the shielding gas rate and the sending powder gas rate used were 16 L min^–1^ and 8 L min^–1^. All samples were prepared using the same LDED parameters, as indicated in Table 3.

### 2.2. Experimental Test

After LDED, the cross-section of the specimen was polished and etched 120 S–150 S using an etching solution (HCl:HNO_3_:H_2_O = 2:1:1). The microstructures, cross-sectional morphologies, and elemental distributions of the discrete and compositional graded samples were observed using a field emission scanning electron microscope (FESEM, Zeiss Ultra Plus, Jena, Germany) equipped with EDS module and a laser confocal microscope (OLYMPUS LEXTOLS4100, Tokyo, Japan). Microhardness distribution from the substrate to the layer surface was measured using a Vickers microhardness tester (EM500-2A) (Shanghai, China) with a load of 500 gf applied for 12 s. Two adjacent test points were marked 100 μm apart.

## 3. Results and Discussion

### 3.1. Microstructure and Microhardness of Various Discrete Samples

**Figure 2:**
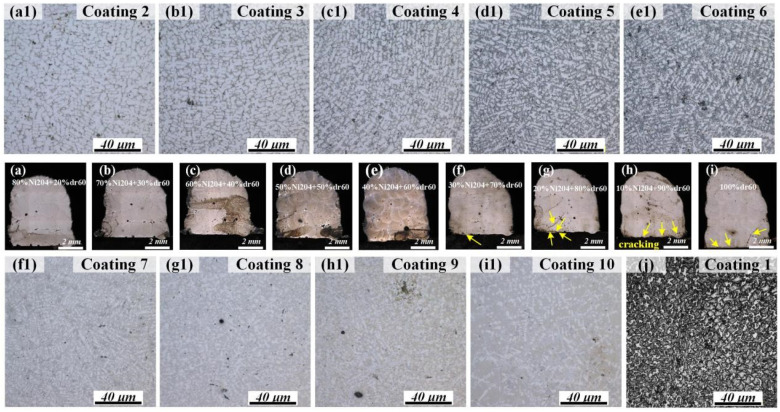
Cross-sectional morphologies of discrete composite samples: (**a**–**j**) samples Ni204_X_dr60_Y_ (0 ≤ x ≤ 80%, 20% ≤ y ≤ 100%); (**a1**–**i1**) enlarged confocal microscope microstructure morphologies corresponding to samples (**a**–**i**).

The cross-sectional morphologies and microstructures of discrete composite samples are presented in Figure 2. As seen in Figure 2a–i, cracks appeared in the interface between the substrate and the Ni204_X_dr60_Y_ composite coating when the dr60 content was more than 70%. In this case, the residual stress at the interface of the composite coating and the substrate is greater than the maximum allowable stress. Figure 2(a1–i1,j) displays an obvious difference in the microstructure. The microstructure of Ni204Xdr60Y resembles that of Ni204 when the Ni204 content is more than 70 wt.%, as shown in Figure 2(a1,b1,j). The composition ranges from 40 to 60 wt.%. Dr60 (Figure 2(c1–e1)) exhibits significant diversity in the microstructure. Clear dendrite structures can still be seen in these samples; however, the grain boundaries exhibit variations. Some novel phases precipitated in the grain boundary, and their contents and sizes increased with the increase in dr60 content. In the dr60 range of 70–90 wt.% (Figure 2(f1–h1)), microstructural characteristics transformed from dendritic structures to deposited dr60 (Figure 2(i1)).

**Figure 3:**
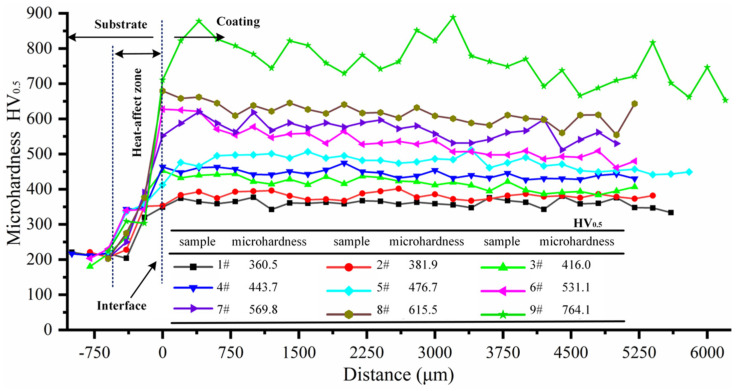
Microhardness corresponding to different Ni204/dr60 mass ratios.

The results of microhardness tests for Ni204_X_dr60_Y_ composite coatings with various mass fraction ratios showed that the dr60 content significantly influenced microhardness distribution in composite coatings (Figure 3). The measurement results indicate that the microhardness of the coatings increased with the dr60 content. The increase in the microhardness of Ni204_X_dr60_Y_ coatings varies approximately linearly with the dr60_Y_ content at levels ranging from 274.6 HV_0.5_ (Ni204_100%_) to 764.1 HV_0.5_ (dr60_100%_). Surprisingly, hardness increased dramatically from 615.5 HV_0.5_ (Ni204_10%_dr60_90%_) to 764.1 HV_0.5_ (dr60_100%_), and Ni204_X_dr60_Y_ coatings exhibited small microhardness fluctuations, with stable microhardness distributions in the coatings. It is worth noting that the differences in the microstructures shown in Figure 2 correspond to the microhardness presented in Figure 3, that is, the microhardness values are associated with the phases that precipitated in the grain boundaries. In order to further investigate the results, the microstructure evolutions of Ni204 → dr60 gradient coatings are analyzed in the subsequent studies.

### 3.2. Characterization of Microstructures and Phase Structures of Graded Samples

**Figure 4:**
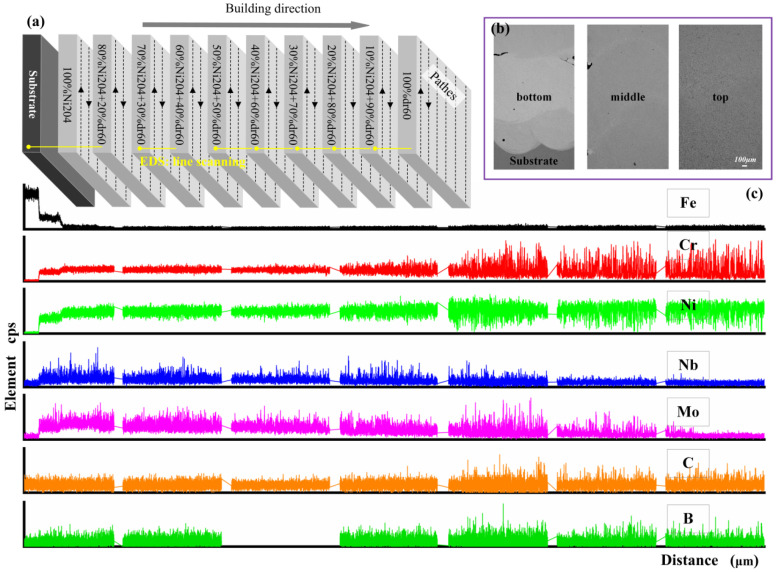
EDS line scan results of the distributions of Nb, Mo, C, B, Fe, Cr, and Ni in Ni204 → dr60 gradient coating. (**a**) EDS line scan test position; (**b**) SEM image of test position; (**c**) EDS results.

Figure 4 shows the distributions of Nb, Mo, B, C, Fe, Cr, and Ni elements in the Ni204 → dr60 gradient coatings. Marginal fluctuations in the Nb and Mo distribution curves from the substrate to the sample end surface become smaller when the dr60 content becomes greater than 50%. In contrast, the fluctuations in C, B, and Cr become larger. EDS line scan results show that many Nb- and Mo-rich phases precipitated in the layers when the content of the added Ni204 was more than 50%, while phases rich in Cr, B, and C precipitated in the layers when the dr60 content was more than 50%. The distribution of the elements in the layers corresponded to the graded materials (Figure 4a). Figure 4b shows that the interface between dissimilar materials has good metallurgical bonding. The concentrations of Nb, Mo, and Cr decreased, while those of B and C increased. Both changes in elements affected the precipitated phases, changing the grain structure and thus affecting the microhardness of the gradient layer. In order to further analyze the influence of these elements, the microstructures of the gradient coatings were anatomized in the following section.

**Figure 5:**
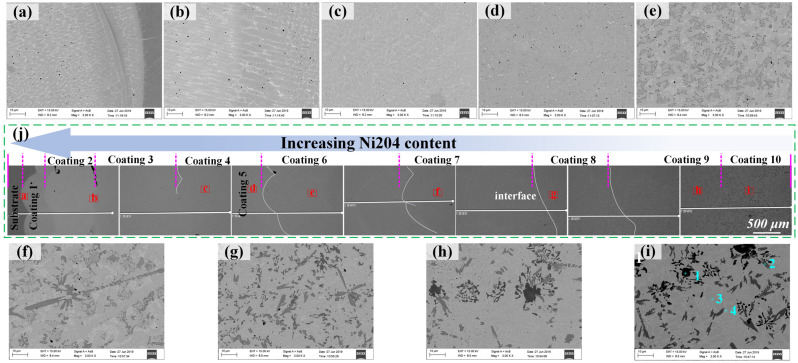
SEM images of cross-sectional microstructures of coatings 1–10. (**a**) Interface between coating 1 and substrate; (**b**) Interface between coating 2 and coating 3, (**c**) Coating 4, (**d**) Coating 5, (**e**) Coating 6, (**f**) Coating 7, (**g**) Coating 8, (**h**) Coating 9, and (**i**) Coating 10.

**Figure 6:**
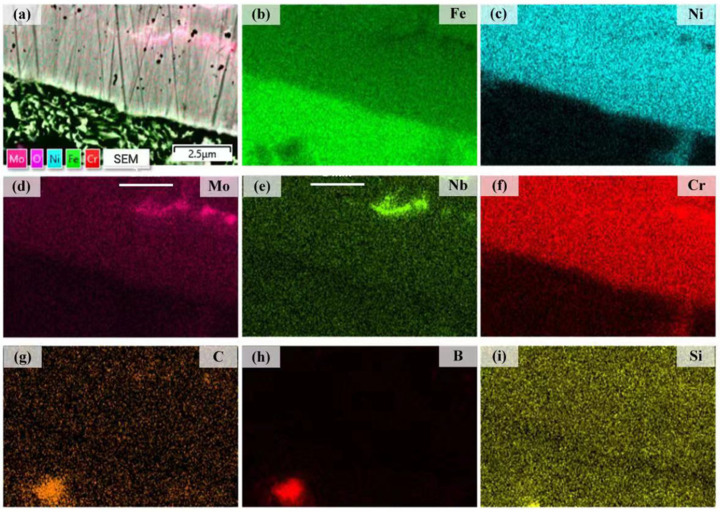
(**a**) SEM image of the interface between substrate and coating 1; (**b**–**i**) EDS maps showing the distributions of Fe, Ni, Mo, Nb, Cr, B, C, and Si.

**Figure 7:**
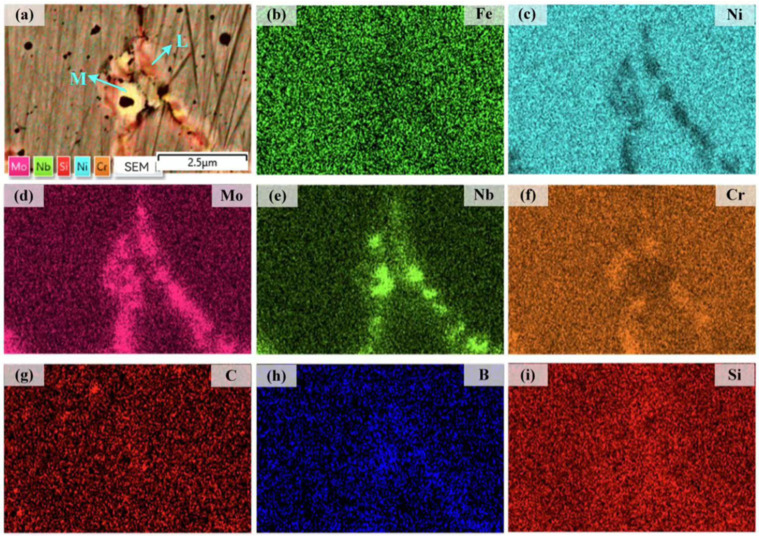
(**a**) SEM image of coating 4; (**b**–**i**) EDS maps showing the distributions of Fe, Ni, Mo, Nb, Cr, C, B, and Si.

From the BSE images in Figure 5a–i, graded microstructure characteristics can be observed in coatings 1–10 of the produced gradient sample. The precipitation phases agglomerated along the grain boundaries, the content and size of precipitated phases increased with the addition of dr60, and the grain boundary subsequently disappeared. Moreover, the interface between the different discrete composite layers rarely showed cracks and pores, as shown in Figure 5j. For coating 1 (Ni204), the cell, columnar, and equiaxed crystals are the main microstructures, as shown in Figure 5a. The long-chain phase (white phase) emerges primarily along grain boundaries and many small bulges are observed in the interface between Ni204 and the substrate, indicating the occurrence of interface diffusion. When the dr60 content is less than 30 wt.%, the grain structure is similar to that of the Ni204 layer (Figure 5b). According to Figure 6 and Figure 7, the white phase mainly contains Nb and Mo as the second phase precipitated at the grain boundaries. Ni, Cr, and Fe are detected in the intragranular zone [Ni, Cr] solid solution. Figure 5c–e shows the initiation of the dramatic change in microstructural characteristics, and long-chain white precipitated phases were gradually replaced with gray lath-shaped phases. With increasing dr60 content, the gray lath-shaped phase varied from a single lath-shaped structure to a network to blocky, and the milk-white phase began to precipitate (Figure 5e). Cr and C atoms were detected in the gray blocky structure phase, indicating that the gray blocky phase is CrC_x_. This phenomenon is related not only to increasing concentrations of B and C but also to the drop in Nb and Mo concentrations. The increase in the content of B and C promoted the precipitation of carbides and borides in the layer, thus reinforcing microhardness. Cr, Nb, and Mo are found mainly at the grain boundary and in the intergranular zones in the course of solidification. Mo and Nb precipitated as the [Nb, Mo] binary phase, Cr combined with C to form a single lath-shaped gray phase CrC_x_, and Cr and C dissolved in the intragranular region near the grain boundaries also precipitated, forming a network phase. With the continuous increase in dr60 content, the black blocky phase gradually superseded the gray network phase. A fresh phase, i.e., the black phase, appears and increases by reuniting to form black blocky phases. At the same time, small milk-white blocky phases appear and gradually increase. At this point, the honeycomb grain structure disappears completely and milk blocky, white, black blocky, and gray blocky phases become uniformly distributed in the matrix, as shown in Figure 5f–i. The results of energy spectrum analysis (Table 4) at points 1–4 in Figure 5i indicate that the black blocky phase at point 1 contains primarily 38.59 au.% Cr, 30.30 au.% B, and 26.00 au.% C atoms, indicating that the phase is a Cr (C, B) ternary phase. The gray phase at point 2 mainly contains 56.29 au.% Cr and 30.61 au.% C atoms (Cr: C = 1.83: 1), indicating that the gray phase is CrC_x_. The 54.38 au.% Ni, 21.53 au.% B, 5.46 au.% Si, and 17.32 au.% C atoms were detected at point 4, indicating that the milk blocky phase is (Ni, Si) (C, B) ternary phase. The matrix areas primarily contain Ni, Cr, and C elements, and the matrix is rich in Ni. The C and B contents increase with the addition of dr60. The CrC_x_ phases were first to precipitate from the Ni–Cr–C–B–Si five-element liquid phase, causing a drop in the concentration of C in the liquid phase. The increased value of the B/C ratio promoted the precipitation of borides, B atoms combined with Cr to form (Cr, B) and dissolved into the CrC_x_ phase to form the Cr (C, B) ternary phase—the black phase. Residues B and C dissolved in the matrix precipitated in the form of multiple (Ni, Si) (B, C) phases. The matrix contained Ni and a small number of Fe, Cr, and other elements, and the solid Ni solution formed via matrix solidification. In the Ni204 → dr60 graded layers, the Mo, Nb, C, and B contents were responsible for the variations in microstructural characteristics and the gradient distribution of microhardness.

Figure 6 illustrates the distributions of Fe, Ni, Mo, Nb, Cr, C, B, and Si elements in the interface between the substrate and coating 1. Nb and Mo are largely distributed in the grain boundaries. Obvious thin bands formed in the interface between the substrate and the Ni204 coating, indicating good metallurgical bonding [31]. However, there were no obvious segregation or diffusion phenomena for Fe, Ni, Mo, Nb, Cr, C, B, and Si elements at the interface.

The distribution of Fe, Ni, Mo, Nb, Cr, C, B, and Si elements in coating 3 is given in Figure 7. Mo, Nb, Cr, B, and C elements are detected in grain boundaries (Figure 5c and Figure 7d–h). The concentrations of these elements at grain boundaries are significantly higher than in the matrix and are precipitated in the form of strengthened phases. Area ‘L’ mainly contains Mo, Cr, and B elements, and area ‘M’ mainly contains Nb, Mo, and B elements, while element C is detected in both areas ‘L’ and ‘M’. With the increase in the concentrations of elements C and B, the grain boundary of the Ni204 coating with [Nb, Mo] as the capital precipitated phase changed to carbide and boride. At this time, the addition of elements C and B promotes the precipitation of Cr, Nb, and Mo in the form of multiple phases at the grain boundaries.

**Figure 8:**
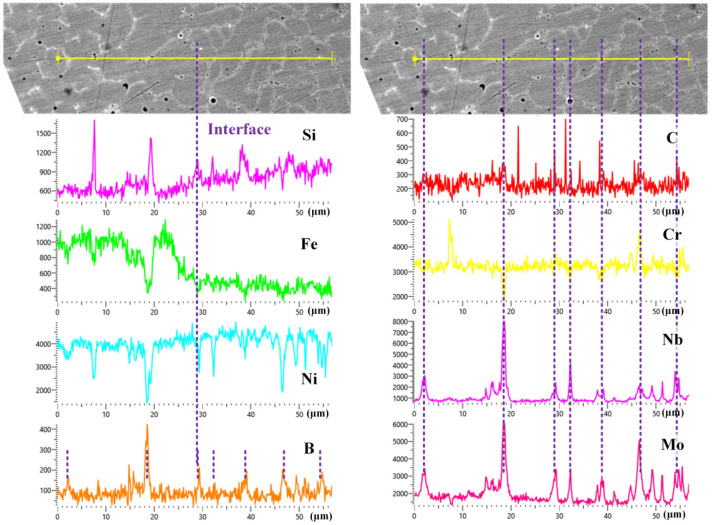
EDS line scan results of Si, Fe, Ni, B, C, Cr, Nb, and Mo at the interface between coating 3 and coating 4.

Figure 8 shows the distributions of Si, Fe, Ni, B, C, Cr, Nb, and Mo at the interface between coating 3 and coating 4. There was good metallurgical bonding quality at the interface; the contents of Si, Fe, and Nb visibly changed, and the gradual content curve indicated the diffusion of elements at the interface. The gradual distribution of elements at the interface reduces the sensitivity of the material to the stress concentration resulting from the sudden change in properties. From the line scan results of elements B, C, Cr, Nb, and Mo at the grain boundaries, one can see that the main elements contained at the grain boundaries are elements Mo, Nb, Cr, B, and C, which are consistent with the results in Figure 5 and Figure 7.

**Figure 9:**
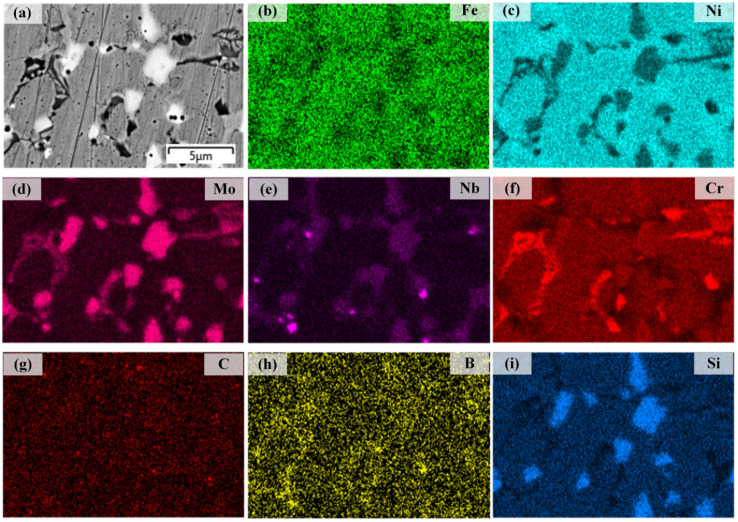
(**a**) SEM image of coating 5; (**b**–**i**) EDS maps showing the distributions of Fe, Ni, Mo, Nb, Cr, C, B, and Si.

**Figure 10:**
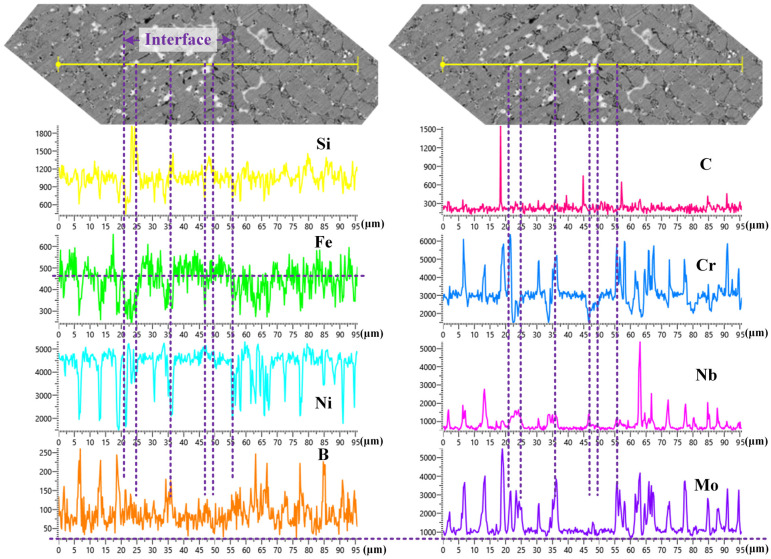
EDS line scan results for Si, Fe, Ni, B, C, Cr, Nb, and Mo at the interface between coating 4 and coating 5.

Figure 9 shows EDS mapping analysis results for elements Fe, Ni, Mo, Nb, Cr, C, B, and Si in coating 5. As seen in Figure 9b,c, the matrix mainly contains Fe and Ni. The milky white phase in Figure 9a largely contains Mo and Nb. EDS line scan results for elements Si, Fe, Ni, B, C, Cr, Nb, and Mo in the interface between coatings 4 and 5 (Figure 10), except elements Nb and Mo, B, C, and Si, are detected in the milky white phase. Elements B, C, and Si dissolved in the [Nb, Mo] phase formed by (Nb, Mo, Si) (C, B). From Figure 9d–h, elements Cr, C, and B are detected in network and blocky black phases, confirming the results in Figure 5.

**Figure 11:**
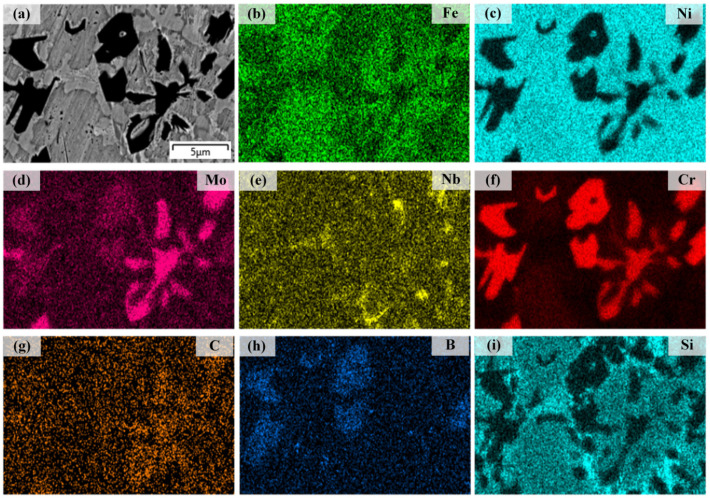
(**a**) SEM image of coating 9; (**b**–**i**) EDS maps showing the distributions of Fe, Ni, Mo, Nb, Cr, C, B, and Si.

Figure 11 shows EDS mapping analysis results for elements Fe, Ni, Mo, Nb, Cr, C, B, and Si in coating 9. The coating was fabricated using 10% Ni204 and 90% dr60. According to Figure 11b, Figure 11c, Figure 11f, and Figure 11i, elements Fe, Ni, Cr, and Si are detected in the matrix. Combined with Figure 5, the matrix is confirmed as [Ni, Fe, Si, Cr]. From Figure 11c,g–i, the gray phase mainly contains Ni and Si; combined with Figure 5, the gray phase is confirmed as (Ni, Si) (C, B). From Figure 11b–i, element Cr is detected in the black phase and there are no Fe, Ni, and Si. The black block phase in the left zone of Figure 11a mainly contains Cr and B, and few Mo and C elements are also detected; combined with Figure 5i, the Cr (C, B) phase is confirmed. The hindering effect of Mo on carbide contributes to the accumulation of Mo around carbides. The network black block phase in the right zone of Figure 11a mainly contains elements Cr, Mo, and C; combined with Figure 5i, the CrC_x_ phase is confirmed. EDS mapping analysis of coating 8 verified the correctness of the phase evolution analysis in Figure 5.

Through phase structure analysis of different coatings and interfaces of the gradient coatings, one can see that with the increase in the contents of elements C and B and the decrease in elements Nb and Mo, the precipitation of equal typical phase structures in grain boundaries and intergranular zones gradually evolves, consistent with the results of the analysis of phase structure evolution of gradient coatings in Figure 5. The change in the structure of the precipitated phase of Cr is the main reason for the change in coating microhardness.

### 3.3. Microhardness of Ni204–dr60 Gradient Composite Materials

**Figure 12:**
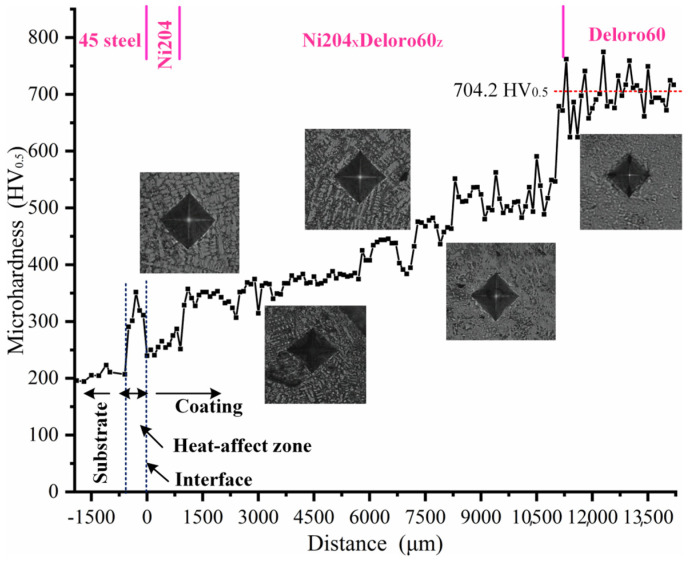
Microhardness of the Ni204–dr60 gradient coating.

Measurement of the microhardness of the Ni204 → dr60 graded coating revealed that the graded coating has a gradient distribution in the depth direction, as shown in Figure 12. Furthermore, and more remarkably, the microhardness values exhibited a sudden increase in the heat-affected zone [24]. The average microhardness of the substrate is about 200 HV0.5, which amounts to heat treatment of the substrate, reinforcing the substrate properties. The variations in the concentrations of elements B and C cause an increase in the precipitation phase at the grain boundary and in the intergranular zones, thus altering the microstructure characteristics and microhardness (Figure 5). Notable positive correlations were observed between microhardness and CrC_x_, Cr (C, B), and (Ni, Si) (C, B) contents. The microhardness of gradient zones arranged in the range established based on the hardness of deposited Ni204 and dr60. Ni, Mo, Nb, B, and C diffused in the interfaces between dissimilar materials. Therefore, the microhardness in the dr60 layer is 704.2HV_0.5_ and drops by 7.8% (60 HV) compared to the discrete dr60 coating. The microhardness showed gradient distribution; however, the value was lower than the value of the corresponding discrete coating (Ni204_X_dr60_Y_, 10% < x, y < 90%), and the diffusions of C, B, Mo, and Nb were primarily responsible for this.

## 4. Conclusions

Graded components of Ni204 → dr60 without visible interface defects and significant microstructure evolution were successfully fabricated. An obvious gradient distribution of hardness was measured in the Ni204 → dr60 gradient coating. Columnar and columnar dendrite crystals were observed near the Ni204 end of the gradient zone (more than 70% of Ni204 added), and an Nb- and Mo-rich precipitated phase existed at the grain boundary and in the intergranular zones. Low hardness was mainly attributed to this microstructure characteristic. However, large numbers of black phase Cr (B, C), gray phase CrC_x_, and milk-white phase (Ni, Si) (C, B) were observed near the dr60 end of the gradient zone (more than 80% of dr60 added). The carbide and boride contents were responsible for the improvement in microhardness in the gradient zone. The reinforced phase of the gradient zone switched from Mo- and Nb-rich precipitated phase → lath-shaped CrC_x_ phase → network-shaped CrC_x_ → network-shaped CrC_x_, block-shaped (Ni, Si) (C, B) phase, and block CrC_x_ and (Ni, Si) (C, B) phase → block CrC_x_, (Ni, Si) (C, B) and Cr (B, C) phase. The changes in the composition of the precipitated phase are mainly responsible for the gradient distribution of microhardness in the graded zone. The graded coating effectively solved the issue associated with crack generation at the interface between dr60 and the substrate. The occurrence of (Fe, Ni) diffusion in various Ni204_X_dr60_Y_ dissimilar materials gives rise to its microhardness, which is slightly lower than that of the corresponding material.

## Figures and Tables

**Table 1:** Chemical compositions of powders and substrate (wt.%).

	C	Si	B	Cr	Mn	Mo	Nb	Ni	Fe
Ni204	≤0.03	0.4		21	-	9	4	Bal.	1.5
dr60	0.71	3.93	2.72	14.93	-	-	-	Bal.	3.56
45^#^ steel	0.42–0.5	0.17–0.37		≤0.25	0.5–0.8	-	-	≤0.25	Bal.

**Table 2:** Chemical compositions of the different layers of the fabricated gradient sample (in wt.%).

Layer No.	Composition	Layer No.	Composition
1–4	100% Ni204	21–24	40% Ni204 60% dr60
5–8	80% Ni204 20% dr60	25–28	30% Ni204 70% dr60
9–12	70% Ni204 30% dr60	29–32	20% Ni204 80% dr60
13–16	60% Ni204 40% dr60	33–36	10% Ni204 90% dr60
17–20	50% Ni204 50% dr60	37–40	100% dr60

**Table 3:** LDED parameters.

Parameters	Laser Power	Scan Speed	Sending Powder Rate	Distance between the Two Layers	Distance between the Adjacent Clad
Value	450 W	5.5 mm s^–1^	0.7 r min^–1^	0.9 mm	0.4 mm

**Table 4:** EDS analysis results of Ni204–dr60 gradient coatings (au. %).

Marked Locations	Fe	Cr	Ni	Nb	Mo	C	B	Si
Point 1	1.47	38.59	3.46	0	0	26.00	30.30	0.18
Point 2	2.79	56.29	9.97	0	0	30.61	0	0.34
Point 3	1.12	11.15	68.25	0	0	16.95	0	2.53
Point 4	1.29	3.72	54.38	0	0	17.32	21.53	5.46

## Data Availability

Not applicable.
